# Inequities in ambulance allocation associated with transfer delay and mortality in acute coronary syndrome patients: evidence from 89 emergency medical stations in China

**DOI:** 10.1186/s12939-022-01777-3

**Published:** 2022-12-16

**Authors:** Siwen Li, Xuejie Dong, Dongmei Li, Hongjuan Zhang, Shuduo Zhou, Mailikezhati Maimaitiming, Junxiong Ma, Na Li, Qiang Zhou, Yinzi Jin, Zhi-Jie Zheng

**Affiliations:** 1 Shenzhen Center for Prehospital Care, 3 Meigang South Street, West Nigang Road, Futian District, Shenzhen, 518025 China; 2 Department of Global Health, School of Public Health, Peking University, 38 Xue Yuan Road, Haidian District, Beijing, 100191 China; 3 Institute for Global Health and Development, Peking University, Beijing, China

**Keywords:** Health resource, Ambulance inequality, Acute coronary syndrome, Prehospital care, Emergency medical service

## Abstract

**Background:**

Allocation of healthcare resources has a great influence on treatment and outcome of patients. This study aimed to access the inequality of ambulance allocation across regions, and estimate the associations between ambulance density and pre-hospital transfer time and mortality of acute coronary syndromes (ACS) patients.

**Methods:**

This cross-sectional study was based on an integrated database of electronic medical system for 3588 ACS patients from 31 hospitals, ambulance information of 89 emergency medical stations, and public geographical information of 8 districts in Shenzhen, China. The primary outcomes were the associations between ambulance allocation and transfer delay and in-hospital mortality of ACS patients. The Theil index and Gini coefficient were used to assess the fairness and inequality degree of ambulance allocation. Logistic regression was used to model the associations.

**Results:**

There was a significant inequality in ambulance allocation in Shenzhen (Theil index: 0.59), and the inequality of inter-districts (Theil index: 0.38) was greater than that of intra-districts (Theil index: 0.21). The gap degree of transfer delay, ambulance allocation, and mortality across districts resulted in a Gini coefficient of 0.35, 0.53, 0.65, respectively. Ambulance density was negatively associated with pre-hospital transfer time (OR = 0.79, 95%CI: 0.64,0.97, *P* = 0.026), with in-hospital mortality (OR = 0.31, 95%CI:0.14,0.70, *P* = 0.005). The ORs of Theil index in transfer time and in-hospital mortality were 1.09 (95%CI:1.01,1.10, *P* < 0.001) and 1.80 (95%CI:1.15,3.15, *P* = 0.009), respectively.

**Conclusions:**

Regional inequities existed in ambulance allocation and has a significant impact on pre-hospital transfer delay and in-hospital mortality of ACS patients. It was suggested to increase the ambulance accessibility and conduct health education for public.

## Introduction

As one of the most dangerous and fatal coronary cardiac diseases, acute coronary syndrome (ACS) is associated with high morbidity and mortality worldwide [1], and has been emerged as one major cause of death in China over the last two decades [2]. Survival and clinical outcomes of ACS patients, especially ST-segment-elevation myocardial infarction (STEMI) patients rely mainly on the early diagnosis and timely medical contact [3, 4]. However, the potential benefits of medical interventions can be largely hampered by pre-hospital delay [5].

Pre-hospital delay is defined as the time interval from patients’ symptom onset to arrival at hospital, which could be divided into “patient delay” (time interval from symptom onset to the decision of seeking medical care) and “transfer delay” (time interval from calling for medical care to arrival at hospital) [6]. It is recommended by guidelines that for ACS patients, the duration from symptom onset to percutaneous coronary intervention (PCI) treatment should be less than 120 minutes [7]. Yet most patients failed to meet this 120-min-criteria: 40% of this “timeout” was due to transfer delay [8].

Emergency medical service (EMS) serves as a key component of pre-hospital care system. By providing rapid diagnosis, early identification, fast treatment, timely alerts to the hospitals, EMS could reduce the transfer delay and improve patient outcomes to a great extent. Over the last decades, the procedure of EMS to treat ACS patients has been well defined, yet it hasn’t resulted in a significant reduction in transfer delay [3, 9]. It is imperative to explore the influential factors in EMS to reduce transfer delay and improve patients’ outcomes.

Allocation of healthcare resources, including personnel, equipment, ambulance, beds in emergency department, have been proven to be one of the major predictors of transfer delay and patient outcome [10]. Many studies indicated that ACS patients living in communities with limited health resources tend to have longer pre-hospital transfer time and worse outcome [11–13]. Although the importance of rational distribution of healthcare resources has been widely recognized, and the system-related factors have been identified [14, 15], the role of ambulance allocation in ACS treatment has remained unclear for a long time until recent studies showing that regional disparities in ambulance density could lead to considerable differences in transfer time and mortality of acute cardiac patients [16]. Despite the raising focus on this field, studies on ambulance allocation in ACS treatment are still small in numbers, and limited in developed countries. As large disparities in health care resources persist between developing countries and developed countries [17], evidence from developing countries with low - and middle-income is needed.

Using an integrated database covering 89 emergency medical stations in Shenzhen, China, this study aimed to access the inequality of ambulance allocation across regions, and estimate the associations between ambulance density and pre-hospital transfer time and mortality of ACS patients.

## Methods

### Data source and study population

This was a cross-sectional study in Shenzhen in 2019. Shenzhen is a special economic city of China with a residential population of approximately 22 million covering 1997.47 km [2]. The EMS in Shenzhen (Shenzhen Emergency Medical Center) was established in 1994, and is now taking the leading position in China with its complete chest pain care system and a 90% automated external defibrillator (AED) coverage.

We used data from an integrated database of Shenzhen hospital electronic medical system for ACS patients, Shenzhen ambulance information, and Shenzhen public population and geographical information. The Shenzhen hospital electronic medical system collected information on numbers, demo characteristics, pre-hospital and in-hospital time records and clinical outcomes of ACS patients from 31 hospitals in Shenzhen. The ambulance information, including numbers of ambulances in each district of Shenzhen was provided by Shenzhen Emergency Medical Center. The public population and geographical information for each district in Shenzhen was collected from Shenzhen Statistics Year Book 2019. We linked these three databases by the code of district, and then derived a two-level integrated database with both the individual-level data on ACS patients and the district-level data on ambulance allocation.

Patients aged over 18 years old with using EMS to arrive at hospital were selected, patients with time records linked to transfer were eligible to be included. From Jan. 1st, 2019 to Dec. 31th, 2019, 3943 out of 14,403 ACS patients aged over 18 years were recorded in the hospital electronic medical system, 355 cases were excluded with missing time records. A total of 3588 ACS cases were included in the final analyses.

Ethics approval and consent to participate this project was approved by the Peking University Health Science Center Institutional Review Board (IRB00001052–21020). Informed consent was obtained from all participants prior to questionnaire administration.

### Measures

#### Outcome measures

The primary outcome variables were analyzed in this study that were: (1) transfer delay time was meaning that the time interval between patients calling EMS and arrival at the emergency department of hospitals and (2) in-hospital mortality.

#### Inequality assessment

The key independent variable was regional ambulance allocation, which was measured by: (1) ambulance density, applied to analyze the amounts of ambulance per kilometer square; (2) Theil index, used to measure the balance of ambulance distribution in eight districts of Shenzhen. Theil Index was usually used as an essential indicator to measure the balance of social resources distribution in one region. It ranges from 0 to 1, where 0 means complete fair in different regions, and 1 means complete unfair. It divided regional resources inequality into two parts: intra-region and inter-region inequality, which can be used to evaluate the contributes of either part to overall inequalities [18]. Theil index also be widely applied to measure healthcare resources allocation. It checks the fairness and inequality of the distribution of resources by checking whether the weight of the area corresponds to the weight of its resource’s allocation [19]; (3) Gini coefficient, used to assess the degree of the inequality in ambulance allocation. The Gini coefficient calculation was according to Lorenz curve and type of Gini coefficient. The degree of disparities in the allocation of ambulance was analyzed by using quantitative indicators, which obtained from data processing and figure area estimation [20]. The value range of Gini coefficient was from 0 to 1. The smaller the value, the smaller the disparities degree, and vice versa [21–23]^.^

#### Covariables

Covariates and potential confounders in this study included patient-level variables and district-level variables. The patient-level variables were age, gender (male, female), heart rate (normal, atrial fibrillation), heart rate (normal, atrial fibrillation), hypertension or not (normal blood pressure, high blood pressure), emergency risk level diagnosed by emergency physicians (low risk, moderate risk, high risk), Killip grade (class grades from I to IV). District-level variables included population density (resident number per square), geographical area.

### Statistical analysis

Basic characteristics of patients were analyzed by descriptive statistics. Continues variables and category variables were presented by mean (SD) and n (%), respectively. The two-sample-T-test and chi-square test were applied to compare differences between patients living in areas with high ambulance density and who living in areas with low ambulance density cases. The Theil index calculation was based on Anand S described in 2010 [24], the formulate was as follows:

$$T=\sum \limits_{i=1}^n{A}_i\log \left(\frac{A_i}{B_i}\right)$$

The Gini index was measured based on the bow area method to analyze the situation of ambulance allocation in districts [25], and Gini coefficient was as follows:

$$G=\sum \limits_{i=1}^n{A}_i{B}_i+2\sum \limits_{i=1}^{n-1}{A}_i\left(1-{C}_i\right)-1$$

The Lorenz curve was plotted with the cumulative percentage geographic area against the cumulative percentage of ambulance resources, transfer time and in-hospital mortality. The Theil index was utilized as the inequality indicator in the regression analysis. Transfer delay was defined as the binary variables by using a 120-minute as cutting point. The Logistic regression models were applied to explore the associations between ambulance allocation and transfer delay and mortality of ACS cases, respectively. All analyses were 2-sided and a *P* value < 0.05 was considered as statistically significant. All analysis were completed by STATA 16.0 (StataCorp, College Station, TX, USA).

## Results

### Characteristics of patients

Data from 3588 ACS patients aged over 18 years old with using EMS (1758 patients live in area with high ambulance density versus 1830 patients live in area with low ambulance density) were included for analysis. Table 1 provided the characteristics of these patients. Among 3588 ACS patients, 326 (9.09%) were death cases. Compared with patients who lived in area with low ambulance density, patient lived in area with high ambulance density were older, more females (20.53% vs 16.89%), less STEMI (1064/1758 vs 1248/1830), with higher emergency risk level (255/1758 vs 121/1830), more arrhythmia (24.69% vs 23.01%), more hypertension (50.74% vs 44.3%), and with lower Killip grade (8.99% vs 11.37%).

**Table 1:** Characteristics of ACS patients (*N* = 3588)

Characteristics	Patients living in areas with high ambulance density (***N*** = 1758)	Patients living in areas with low ambulance density (***N*** = 1830)	Total (***N*** = 3588)	***P-value***
**Age, mean (SD)**	58.43(13.81)	56.85(13.82)	–	< 0.001
**Gender, n (%)**				0.005
Male	1397(79.47)	1521(83.11)	2918(81.33)	
Female	361(20.53)	309(16.89)	670(18.67)	
**Death cases, n (%)**	132(7.51)	194(10.60)	326(9.09)	0.001
**Type of disease, n (%)**				< 0.001
STEMI	1064(60.52)	1248(68.20)	2312(64.44)	
NSTEMI	488(27.76)	384(20.98)	872(4.30)	
UA	206(11.72)	198(10.82)	404(11.26)	
**Emergency risk level, n (%)**				< 0.001
Low	1177(66.95)	1540(84.15)	2717(75.72)	
Medium	326(18.54)	169(9.23)	495(13.80)	
High	255(14.51)	121(6.61)	376(10.48)	
**Heart rate, n (%)**				0.237
Normal	1324(75.31)	1409(76.99)	2733(76.17)	
Arrhythmia	434(24.69)	421(23.01)	855(23.83)	
**Hypertension, n (%)**				< 0.001
Yes	892(50.74)	813(44.43)	1705(47.52)	
No	866(49.26)	1017(55.57)	1883(52.48)	
**Killip grade, n (%)**				< 0.001
0	1412(80.32)	1399(76.45)	2811(78.34)	
1	106(6.03)	102(5.57)	208(5.80)	
2	36(2.05)	27(1.48)	63(1.76)	
3	46(2.62)	94(5.14)	140(3.90)	
4	158(8.99)	208(11.37)	366(10.20)	

### Inequality and gap degree in ambulance allocation, transfer delay and in-hospital mortality

**Fig. 1:**
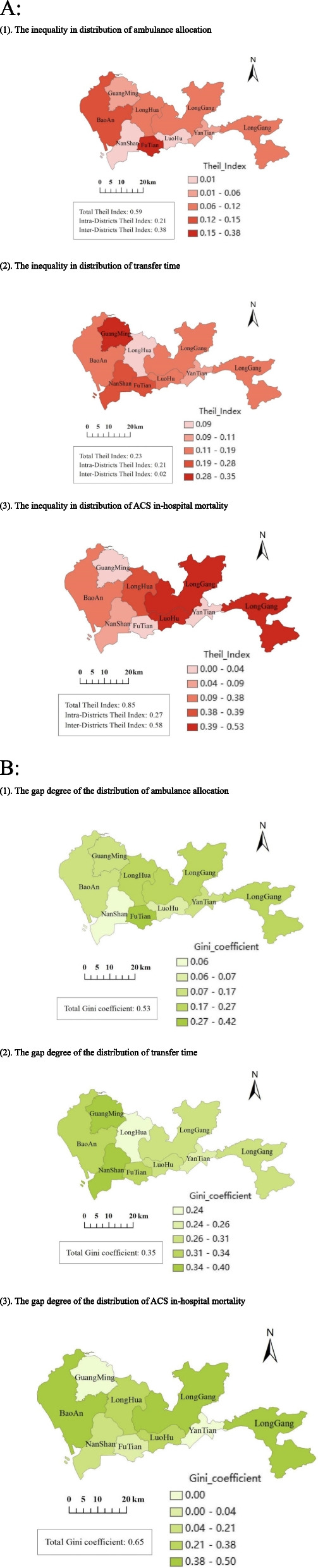
The inequalities and gap degree in distribution of ambulance allocation, transfer time, and mortality across eight districts of Shenzhen. **A** The inequality in distribution across geography measured by Theil index. (1): The inequality in distribution of ambulance allocation. (2): The inequality in distribution of transfer time. (3): The inequality in distribution of ACS in-hospital mortality. **B** The gap degree of the distribution across geography measured by Gini coefficient. (1): The gap degree of the distribution of ambulance allocation. (2): The gap degree of the distribution of transfer time. (3): The gap degree of the distribution of ACS in-hospital mortality

Figure 1 showed the inequalities and gap degree in distribution of ambulance allocation, transfer time and in-hospital mortality across eight districts of Shenzhen in 2019. There was a great inequality in ambulance allocation across districts (Theil index: 0.59, Gini coefficient: 0.53), among which the inequality of inter-districts (Theil index: 0.38) was greater than that of intra-districts (Theil index: 0.21). The greatest inequality in ambulance allocation was found in Futian (Theil index: 0.38, Gini coefficient: 0.42), while Nanshan (Theil index: 0.01, Gini coefficient: 0.06) and Luohu (Theil index: 0.01, Gini coefficient: 0.07) had the lowest inequality in ambulance allocation.

Figure 1A (2) and B (2) revealed the disparities in pre-hospital transfer time (Theil index: 0.23, Gini coefficient: 0.35). The intra-districts transfer time disparity was higher (Theil index: 0.21) than that of inter-districts disparity (Theil index: 0.02). Fig. 1A (3) and B (3) presented the disparities of ACS in-hospital mortality. The results expressed a significant inequality with highly gap degree of overall mortality (Theil index: 0.85, Gini coefficient: 0.65). The inter-district mortality disparity (Theil index: 0.58) was significantly higher than that of intra-district (Theil index: 0.27).

**Fig. 2:**
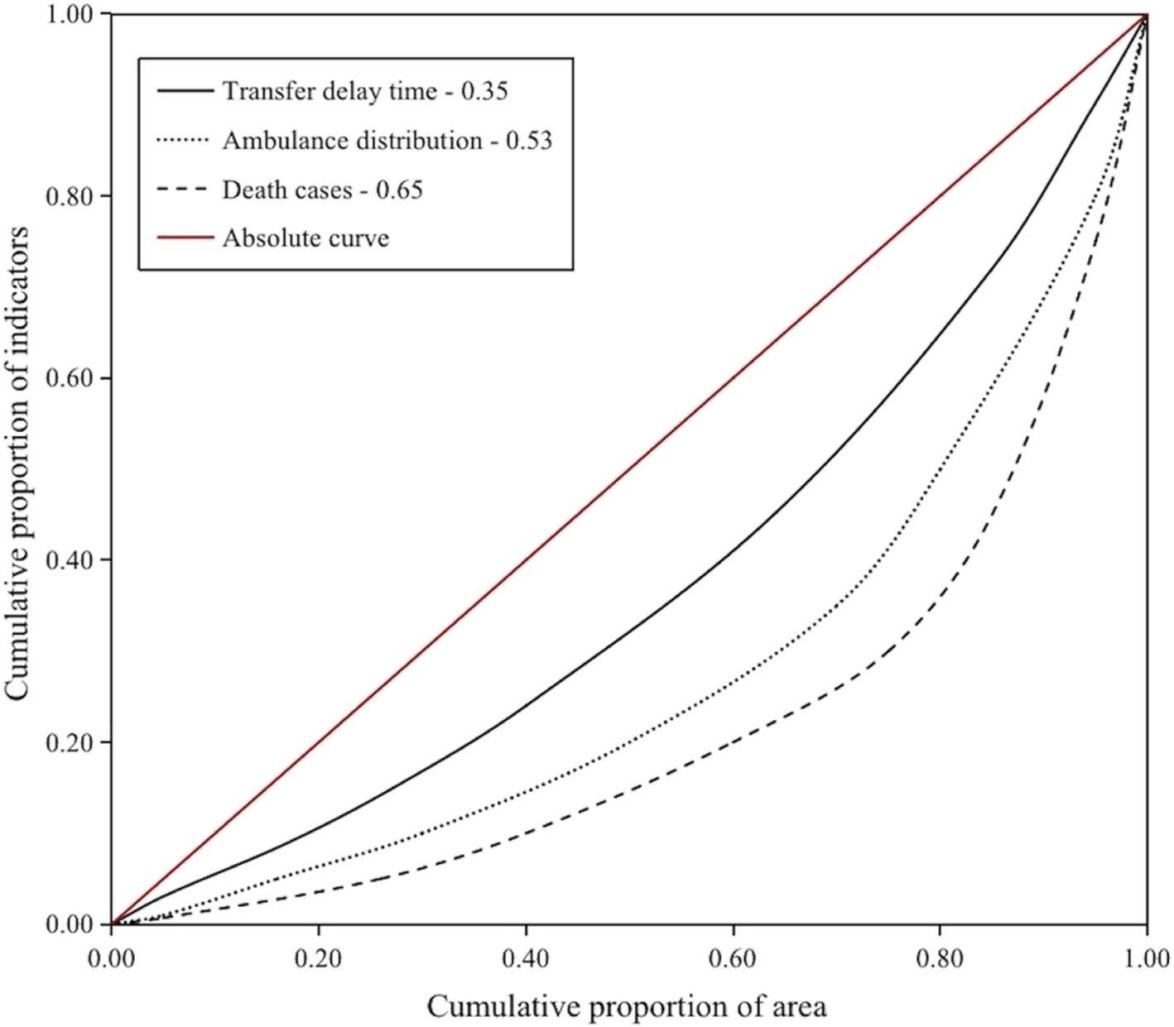
The Lorenz curve of the distribution of ambulance allocation, transfer time, and in-hospital mortality across geography

Figure 2 presented Lorenz curves with cumulative proportion of geographic area on the x-axis and cumulative proportion of indicators on the y-axis. The inequality degree in distribution of transfer time, ambulance allocation, and mortality resulted in a Gini coefficient of 0.35, 0.53, 0.65, respectively.

### Association between ambulance allocation inequality with transfer delay and in-hospital mortality for ACS patients

Table 2 showed the odds ratios of multivariate logistic regressions. In the Model a, ambulance density was negatively associated with transfer delay (OR = 0.79, 95%CI:0.64,0.97, *P* = 0.026). The OR of ambulance density in in-hospital mortality was 0.31 (95%CI:0.14,0.70, *P* = 0.005) (Model b). In addition, we found that the positive association between Theil index and transfer delay in the Model c (OR = 1.09, 95%CI:1.01,1.10, *P* < 0.001). Similar pattern was also observed for in-hospital mortality in the Model d (OR = 1.80, 95%CI:1.15,3.15, *P* = 0.009).

**Table 2:** Association between ambulance allocation with transfer delay and mortality of ACS patients

	Transfer delay			In-hospital mortality		
	OR	95% CI	*P*-value	OR	95% CI	*P*-value
**Ambulance density** ^**a, b**^	0.79	(0.64, 0.97)	0.026	0.31	(0.14, 0.70)	0.005
**Theil index** ^**c, d**^	1.09	(1.01, 1.10)	< 0.001	1.80	(1.15, 3.15)	0.009

Transfer delay was defined as a binary variable by using a 120-minute as a cutting point

^a^Logistic regression model was to measure the effect of ambulance density on transfer delay: Covariates for include district-level variables (regional geographic area, regional population density) and patient-level variables (age, gender, emergency risk level, Killip grade, heart rate);

^b^Logistical regression model was to measure the effect of ambulance density on mortality: Covariates include district-level variables (regional population density), system-level variable (transfer delay time) and patient-level variables (age, gender, emergency risk level, Killip grade, heart rate)

^c^Logistical regression model was to measure the effect of Theil index on transfer delay: Covariates include district-level variables (regional population density), system-level variable (transfer delay time) and patient-level variables (age, gender, emergency risk level, Killip grade, heart rate)

^d^Logistical regression model was to measure the effect of Theil index on mortality: Covariates include district-level variables (regional population density), system-level variable (transfer delay time) and patient-level variables (age, gender, emergency risk level, Killip grade, heart rate)

## Discussion

In this cross-sectional study based on an integrated database of 8 districts, 89 EMS centers, and 31 hospitals in Shenzhen, we found significant inequalities with obvious gap degree in ambulance allocation, pre-hospital transfer time and clinical outcomes of ACS patients across districts of Shenzhen. Multilevel regression analysis revealed that regional ambulance density and balance of ambulance distribution were important factors associated with pre-hospital transfer delay and in-hospital mortality of ACS patients. Our findings were consistent with previous studies in which regional disparities in ambulance density could lead to considerable differences in transfer time and mortality of acute cardiac patients [16, 26], and provided evidence from developing countries to show the importance of optimizing the allocation of emergency medical resources.

Ambulance density is a modifiable factor to reduce the pre-hospital transfer delay. Our findings showed that the association between ambulance density and transfer delay was negatively significant. Increasing ambulance density could improve the treatment efficiency of EMS system by solving the crowding in local ambulance demand and influencing the behavior of patients. With increasing demand of EMS, ambulance crowding is a common barrier to timely transfer of ACS patients [27], which could also cause a series of delay in ambulance response and returning [28], and moreover, may hamper the willingness of patients to call for ambulance, especially for patients with time-sensitive illnesses [29]. Ambulance use can reduce the pre-hospital delay to at least half an hour in regardless of the location of the patients [30], however, limitation in health resources could influence the utilization of ambulance among patients with ACS [31]. Studies showed that instead of calling and waiting for ambulance, patients living in regions with inadequate resources preferred to go to hospitals by self-transportations [32]. Consistent with Nogueira et al., our findings indicated that modifying ambulance density was a feasible way to prompt EMS efficiency [33]. It is warranted to standardize the transport protocols, increase the availability and accessibility of ambulance in order to shorten the pre-hospital delay.

Our results showed that higher ambulance density was associated with lower ACS in-hospital mortality, which may be mainly attributable to the increased number of physician and nurses onsite. The presences of physician in the ambulance can improve the clinical outcomes of cardiac disease patients [34, 35]. On the one hand, ambulance crews are the first medical contactors of patients calling EMS, they can provide timely electrocardiogram (ECG) and cardiopulmonary resuscitation (CPR), communicate with the hospitals, and transmit the electronic ECG report to the emergency departments at the same time [36]. On the other hand, the coordination care of pre-hospital and emergency department can fasten the triage of ACS patients, and reduce the time for interventions to patients with high clinical risks, and therefore improve the short-term and long-term outcomes of ACS patients [37]. However, the benefits of pre-hospital care services may be not obvious in some settings of China. Patients’ lack of recognition of ACS, or uncontrolled self- medication before calling for help could all lead to a delay of disease assessment in the ambulance [38]. In parallel to increasing ambulance accessibility, community health education is also warranted.

Our findings revealed an unbalance distribution with obvious gap in ambulance allocation across eight districts in Shenzhen by calculating Theil index and Gini coefficient. The government of Shenzhen city provides sufficient health expenditure to each district. However, there are large disparities in healthcare decentralization across districts [39, 40], giving rise to the large inequalities and disparities in inter-regional ambulance allocation, which accounted for 64% of the overall ambulance allocation inequality. Regional ambulance allocation unfairness with highly degree could also be caused by the different roles of the district governments with respect to spending on emergency medical care [41], and the differences in development priorities to districts polities [42].

Finally, we found that the unfairness distribution and high degree of inequalities in the allocation of ambulance were associated with prolonged transfer time and higher ACS mortality, which is compatible with other studies [26]. Clinical outcome of ACS patients could be affected by both patient-related and system-related factors [43–45]. Our study adjusted for patient-related factors to focus on system variables, indicating that equal distribution of ambulance allocation can contribute to an improvement in health system performance and clinical outcomes of ACS patients.

### Limitation

There are some limitations in the current study. First, the data of our study were obtained in 2019, whereas during recent years, the number of ambulances might have increased in Shenzhen and the disparities in spatial distribution of ambulance now may be different. Secondly, though we included covariates and potential confounders of both patient-level and district-level, unmeasured confounding factors such as regional traffic environment and its medical care ability may have influenced the outcomes. Lastly, we enrolled ACS cases from 89 EMS centers and 31 hospitals in Shenzhen city with complete registry, which might limit the generalizability of our findings.

## Conclusion

Inequities in regional ambulance allocation has a significant impact on pre-hospital transfer delay and in-hospital mortality of ACS patients. Policy interventions and suggestions in increasing ambulance accessibility and health education for public should be considered for future development.

## Data Availability

The datasets used and analyzed during the current study are available from the corresponding author on reasonable request。.

## Abbreviations

ACS: Acute Coronary Syndrome
STEMI: ST-segment-elevation Myocardial
PCI: Percutaneous Coronary Intervention
EMS: Emergency Medical Service; Shenzhen Emergency Medical Center: The EMS in Shenzhen
AED: Automated External Defibrillator
NSTEMI: Non-ST Elevation Myocardial Infarction
UA: Unstable Angina
EMS: Emergency Medical Service
SBP: Systolic Blood Pressure
DBP: Diastolic Blood Pressure
